# Hearing and dementia: from ears to brain

**DOI:** 10.1093/brain/awaa429

**Published:** 2020-12-22

**Authors:** Jeremy C S Johnson, Charles R Marshall, Rimona S Weil, Doris-Eva Bamiou, Chris J D Hardy, Jason D Warren

**Affiliations:** 1 Dementia Research Centre, Department of Neurodegenerative Disease, UCL Queen Square Institute of Neurology, University College London, London, UK; 2 Preventive Neurology Unit, Wolfson Institute of Preventive Medicine, Queen Mary University of London, London, UK; 3 Movement Disorders Centre, Department of Clinical and Movement Neurosciences, UCL Queen Square Institute of Neurology, University College London, London, UK; 4 Wellcome Centre for Human Neuroimaging, UCL Queen Square Institute of Neurology, University College London, London, UK; 5 UCL Ear Institute and UCL/UCLH Biomedical Research Centre, National Institute for Health Research, University College London, London, UK

**Keywords:** hearing, dementia, Alzheimer’s disease, frontotemporal dementia, Lewy body disease

## Abstract

The association between hearing impairment and dementia has emerged as a major public health challenge, with significant opportunities for earlier diagnosis, treatment and prevention. However, the nature of this association has not been defined. We hear with our brains, particularly within the complex soundscapes of everyday life: neurodegenerative pathologies target the auditory brain, and are therefore predicted to damage hearing function early and profoundly. Here we present evidence for this proposition, based on structural and functional features of auditory brain organization that confer vulnerability to neurodegeneration, the extensive, reciprocal interplay between ‘peripheral’ and ‘central’ hearing dysfunction, and recently characterized auditory signatures of canonical neurodegenerative dementias (Alzheimer’s disease, Lewy body disease and frontotemporal dementia). Moving beyond any simple dichotomy of ear and brain, we argue for a reappraisal of the role of auditory cognitive dysfunction and the critical coupling of brain to peripheral organs of hearing in the dementias. We call for a clinical assessment of real-world hearing in these diseases that moves beyond pure tone perception to the development of novel auditory ‘cognitive stress tests’ and proximity markers for the early diagnosis of dementia and management strategies that harness retained auditory plasticity.

## Teaser

Epidemiological data indicate that mid-life hearing loss is a modifiable risk factor for dementia, with potentially major diagnostic and therapeutic implications. Johnson *et al*. explore why realising this potential requires understanding hearing as a complex brain function that is especially vulnerable to neurodegeneration.

## Introduction: scope and nature of the problem

Hearing impairment in later life is a major clinical issue and a leading association of cognitive decline (Gates and Mills, 2005; Lin *et al.*, 2011; Loughrey *et al.*, 2018), presenting significant potential opportunities for dementia diagnosis, treatment and prevention (Dawes *et al.*, 2015; Taljaard *et al.*, 2016; Livingston *et al.*, 2017). But how are hearing impairment and dementia related? Hearing loss of any cause tends to limit social engagement and quality of life (Graydon *et al.*, 2019), amplifies the effects of cognitive impairment and may confound or delay diagnosis of dementia (Panza *et al.*, 2015; Wayne and Johnsrude, 2015). Conversely, diagnosis of hearing loss and compliance with hearing aids are hindered by cognitive impairment (Dawes *et al.*, 2015). There may, however, be a more fundamental pathophysiological basis for the association: hearing is a complex cognitive function that, alongside other cognitive functions, is directly vulnerable to the pathophysiological processes that cause dementia (Wayne and Johnsrude, 2015; Hardy *et al.*, 2016).

**Figure 1:**
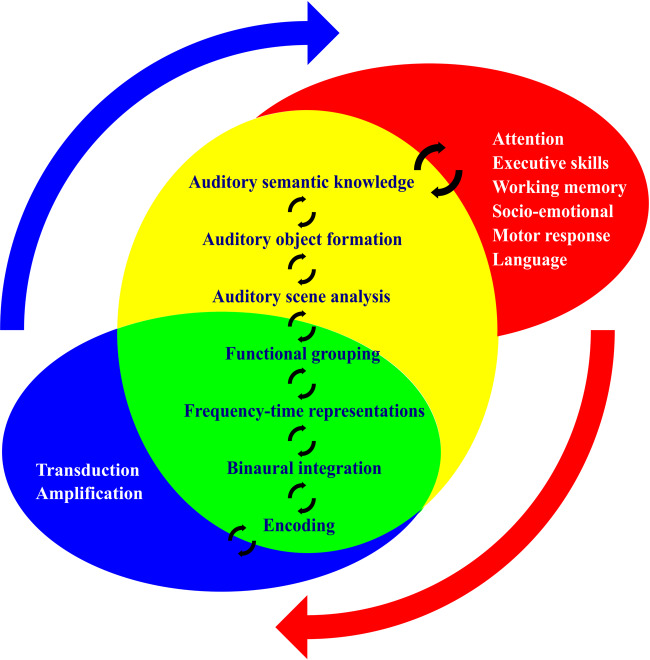
**Processes and interactions in ‘peripheral’ and ‘central’ hearing.** The functional organization of the auditory processing hierarchy and the interplay of hearing with more general cognitive functions. Ellipses indicate the broad domains of peripheral hearing (blue; anatomically, the peripheral hearing apparatus which receives incoming sounds, cochlea and auditory nerve), precognitive auditory processing (green; chiefly the auditory brainstem), auditory cognition (yellow; auditory cortex and its cerebral connections) and general cognitive functions (red; see Fig. 2 for neuroanatomy). Listed within the ellipses are some key stages in the analysis of auditory information: ‘peripheral’ and ‘central’ hearing processes lie on a functional and anatomical continuum, with reciprocal connections between successive processing stages (black arrows). This organization implies that pathologies (such as neurodegenerative proteinopathies) predominantly targeting auditory cognitive (and general cognitive) processing stages may have cascading effects at other processing stages. Certain additional functional properties that operate across auditory processing stages, such as non-linear signal coding and plasticity, are likely to be particularly vulnerable to the effects of neurodegenerative pathologies (see text). External red and blue arrows here signify general mechanisms by which hearing dysfunction of any cause may promote cognitive decline, and the converse; these mechanisms are likely to be mutually reinforcing and may additionally compound more specific effects of auditory brain dysfunction, with the potential to establish pathophysiological ‘vicious cycling.’

**Figure 2:**
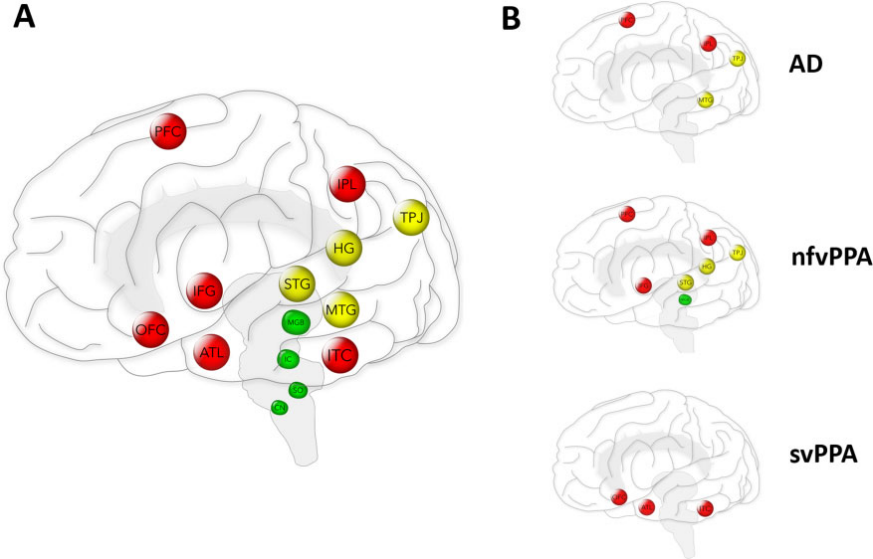
**The auditory brain in health and neurodegenerative disease.** (**A**) Major anatomical regions that mediate the processes underpinning hearing (Fig. 1) as spheres overlaid in a left lateral view of the brain. These regions are anatomically and functionally linked into large-scale, distributed networks. The colour convention follows that in Fig. 1 (green, precognitive auditory processing in brainstem pathways, enclosed by the grey filled outline; yellow, auditory cognition in auditory cortices; red, general cognitive processes in connected cerebral regions); note, however, that there is no simple, one-to-one correspondence between particular brain regions and individual ‘tiers’ of the processing hierarchy outlined in Fig. 1. Brain regions are designated as follows: ATL = antero-mesial temporal lobe (also encompassing amygdala and hippocampus); CN = cochlear nucleus (ventral and dorsal); HG = Heschl’s gyrus (medial portion contains primary auditory cortex); IC = inferior colliculus; IFG = inferior frontal gyrus (closely associated with insular cortex, deep to the cerebral surface); IPL = inferior parietal lobe; ITC = inferior temporal cortex; MGB = medial geniculate body; MTG = middle temporal gyrus; OFC = orbitofrontal cortex; PFC = prefrontal cortex; SO = superior olive (its main projection in the lateral lemniscus has several additional, small associated nuclei); STG = superior temporal gyrus; TPJ = temporo-parietal junctional cortex. Also shown in grey filled outline is the cingulate gyrus, projected from the medial surface of each cerebral hemisphere: this signifies linked deep medial prefrontal and parietal cortices that also participate importantly in integrative and modulatory cognitive processes relevant to hearing. (**B**) Key components of the brain networks implicated in hearing that are also predominantly targeted in representative neurodegenerative proteinopathies. These patterns of brain degeneration anticipate the differential involvement of particular auditory functions and therefore distinctive functional hearing profiles or ‘auditory phenotypes’ of these disorders (see text and Table 1). Although the neuroanatomical patterns shown correspond to the distribution of most severe regional brain atrophy in each disease, dysfunction predates atrophy and additional connected brain regions may also be implicated in the pathogenesis of auditory symptoms. AD = typical Alzheimer’s disease; nfvPPA = non-fluent agrammatic variant primary progressive aphasia; svPPA = semantic variant primary progressive aphasia.

Recent studies addressing the link between hearing impairment and dementia have focused predominantly on audiometric pure tone detection, the ability to detect quiet sounds (Lin *et al.*, 2011; Loughrey *et al.*, 2018). However, most natural auditory environments or ‘scenes’ comprise mixtures of sounds that change over time, and listening—perception and understanding of sounds—is a highly active cognitive process (Bendixen, 2014) (Fig. 1). Consider, for example, the everyday scenario of following a conversation in a crowded room. After substantial ‘pre-cognitive’ processing in the auditory brainstem (Cope *et al.*, 2015), the incoming auditory signal must be deconstructed (by ‘auditory scene analysis’) (Goll *et al.*, 2012*a*; Golden *et al.*, 2015*c*; Hardy *et al.*, 2016) into discrete and stable percepts or ‘auditory objects’ corresponding to voices and speech features, separate from background noise (Griffiths and Warren, 2004; Goll *et al.*, 2010*b*). Such auditory objects must be matched to stored representations and expectations to achieve recognition and ultimately, an appropriate behavioural response. These processes collectively constitute ‘auditory cognition’ (Fig. 1) and depend critically on neural computations in auditory cortical and linked processing networks: the auditory brain (Fig. 2).

Evidence that neurodegenerative pathologies target the auditory brain and produce ‘central’ hearing deficits disproportionate to any peripheral hearing loss was first produced some time ago (Kurylo *et al.*, 1993; Strouse *et al.*, 1995). More recently, a diverse array of ‘central’ auditory deficits has been described in these diseases (Mahoney *et al.*, 2011; Rohrer *et al.*, 2012; Fletcher *et al.*, 2015, Golden *et al.*, 2015*c*; Grube *et al.*, 2016; Hardy *et al.*, 2016; Eversfield and Orton, 2019; Jafari *et al.*, 2020), ranging widely beyond ‘deafness’ (impaired sound detection) to encompass altered auditory perception, understanding and behavioural responses, with far-reaching consequences for hearing function in daily life. To date, however, the role of the auditory brain in linking hearing impairment to cognitive decline has been largely overlooked.

Here we argue that the auditory brain is integral to the development and expression of hearing impairment in dementia. Our case rests on three interwoven lines of evidence: the structural and functional characteristics of auditory brain organization targeted by neurodegenerative diseases; the known extensive interplay between so-called ‘peripheral’ and ‘central’ hearing mechanisms; and mounting data on auditory cognitive dysfunction as a prominent, early and specific manifestation of canonical dementia syndromes. We propose a roadmap for future work directed towards developing novel auditory cognitive tests, biomarkers and therapies.

## The auditory brain: structural and functional substrates for neurodegeneration

The auditory system has evolved to allow adaptive behavioural responses to complex, dynamic acoustic environments (Griffiths *et al.*, 2001; Pickles, 2015). However, its structural and functional characteristics confer specific vulnerabilities to neurodegenerative pathologies.

Anatomically, the hierarchy of auditory processing relays and in particular the large-scale cerebral networks that process sound information (Fig. 2) are highly distributed. The spread of pathogenic proteins in neurodegenerative dementias (Fig. 2) targets these networks rather than the peripheral organs of hearing. Though histopathological data remain limited, neurodegenerative pathologies may preferentially involve auditory association cortex and cortico-cortical projections rather than primary sensory cortex (Esiri *et al.*, 1986; Lewis *et al.*, 1987), thereby striking the integrative mechanisms that are most critical for auditory object analysis.

Accurate auditory signal transduction (for example, during spatial hearing or speech perception) depends on precise integration of frequency-based (spectral) and time-based (temporal) information (Griffiths *et al.*, 2001; Bizley *et al.*, 2009): any pathology that damages relevant neural circuits is likely to disrupt such processing early in its course. As the auditory signal passes up the processing hierarchy, it is transformed non-linearly such that it is no longer a direct replica of the incoming signal encoded at the periphery (Wang, 2007; Gaucher *et al.*, 2013); due to the intrinsically temporal nature of sound, this transformation of auditory information is particularly evident in the time domain and supports the extraction of invariant auditory object features and cross-modal integration. The resulting percept is normally robust to noisy variations in the sensory signal; however, its non-linear nature means that even small perturbations of neural circuit function due to neurodegenerative disease may have disproportionately large perceptual and behavioural consequences.

Two additional, related guiding principles of auditory system operation that are critical for adaptive functioning in complex, dynamic auditory environments are functional plasticity and reciprocity. Reciprocity is mediated by recursive, afferent-efferent feedback that supports auditory change detection and top-down tracking of behaviourally relevant sound sources (Shamma and Micheyl, 2010; Zion Golumbic *et al*., 2013), as well as predictive decoding and ‘filling-in’ of ambiguous and varying auditory inputs, such as degraded speech (Malmierca, 2014; Simon, 2015; Donhauser and Baillet, 2020) (Fig. 1). Plasticity (for example, perceptual learning of degraded speech) (Hardy *et al.*, 2018) enables dynamic neural adaptation to auditory experience.

These functional principles are evident throughout the auditory system (Russo *et al.*, 2005; Barascud *et al.*, 2016; Guinan, 2018) and are highly sensitive to synaptic neurochemical (particularly cholinergic) modulation, especially under challenging listening conditions (Dhanjal *et al.*, 2013; Kuchibhotla *et al.*, 2017; Minces *et al.*, 2017). They are therefore potentially highly susceptible to neurodegenerative pathologies that disrupt synaptic and neurotransmitter pathway integrity. Moreover, the characteristics of non-linear stimulus coding, extensive efferent regulation of afferent pathways and pervasive plasticity (though not specific to audition) are much more marked in the auditory system than in other sensory systems, notably vision (King and Nelken, 2009). Impaired functional adaptation of auditory brainstem pathways has perceptual consequences in patients with mild cognitive impairment (Bidelman *et al.*, 2017), suggesting that indices of auditory plasticity may be sensitive and dynamic markers of neurodegenerative pathologies.

## ‘Peripheral’ and ‘central’ hearing: a false dichotomy and a double hit

The anatomical and functional interactions of auditory processing stages (Figs 1 and 2) suggest that any sharp distinction between ‘peripheral’ and ‘central’ hearing is likely to be a false dichotomy. Pure tone audiometry (PTA), the mainstay of standard clinical audiological assessment, is generally interpreted as an index of ‘peripheral’ (cochlea and auditory nerve) hearing. However, PTA thresholds are affected by attention (Musiek *et al.*, 2017), executive function (Gates *et al.*, 2010) and brainstem pathologies that do not directly involve the cochlea (Cope *et al.*, 2015), reflecting the known role of top-down influences on cochlear sensitivity (Terreros and Delano, 2015). Furthermore, PTA does not fully predict ability to hear speech in noise (the principal hearing complaint of older listeners) (Anderson *et al.*, 2011; Guest *et al.*, 2018; Holmes and Griffiths, 2019). Conversely, ‘central’ hearing functions that rely on high-fidelity signal coding at brainstem level (such as speech intelligibility) are tuned by efferent synaptic functional adaptation at the cochlea (Pressnitzer *et al.*, 2008) and auditory agnosia is modulated by peripheral hearing loss (Coebergh *et al.*, 2020). Neurodegenerative diseases that principally involve cortical and subcortical pathways may therefore significantly impact hearing functions canonically attributed to the peripheral sense organs; indeed, elevated PTA thresholds have recently been documented in the non-fluent agrammatic variant of primary progressive aphasia (nfvPPA), a primary cortical degeneration (Hardy *et al.*, 2019). On the other hand, anatomical involvement of subcortical auditory relays by neurodegenerative pathology does not necessarily lead to a perceptual deficit (Hughes *et al.*, 2014).

Moreover, neurodegenerative diseases typically target the ageing brain, and healthy ageing itself affects multiple stages of auditory processing, ranging from cochlea to cortex (Bendixen, 2014; Bidelman *et al.*, 2014; Roth, 2015; Henry *et al.*, 2017; Zhao *et al.*, 2019). Some of these effects (in particular, degeneration of synapses between inner hair cells and auditory nerve fibres) are undetectable or ‘hidden’ on standard PTA and may therefore be underestimated (Wu *et al.*, 2019); other effects (such as attentional suppression of irrelevant sensory information) may only emerge under challenging listening conditions or for particular tasks, such as tracking fine-grained temporal information in speech (Henry *et al.*, 2017). Increased cognitive effort and engagement of task-relevant capacities (in auditory cortex or executive control systems) may compensate to a degree for the widespread effects of ageing on auditory signal processing (Profant *et al.*, 2015; Meister *et al.*, 2016; Glick and Sharma, 2017; Bidelman *et al.*, 2019); however, if compensatory mechanisms are compromised by neurodegenerative pathology, this ‘double hit’ may cause hearing loss to become functionally significant. Such decompensation would be relatively more likely under adverse listening conditions. In this context, neurodegenerative effects on auditory brain function might act as ‘proximity makers’ for incipient, more generalized cognitive decline.

## Major dementias have diverse auditory phenotypes

The neurodegenerative diseases that cause canonical dementia syndromes have specific profiles of large-scale, cortico-subcortical network involvement, determined by the patterns of spread of pathogenic proteins (Seeley *et al.*, 2009; Warren *et al.*, 2013) (examples in Fig. 2). These pathologies have correspondingly diverse clinical phenotypes including prominent auditory cognitive deficits (Table 1).

**Table 1:** Auditory phenotypes of some major dementia syndromes

Syndrome	Core clinical features	Key auditory symptoms	Auditory deficits^a^	Proposed auditory diagnostic test^b^	Pathological neuroanatomy^c^
**Alzheimer’s disease**					
Typical	Episodic/topographical memory loss, parietal deficits	Difficulty tracking sound sources/information in busy acoustic environments, auditory disorientation, difficulty understanding less familiar accents, auditory agnosia, increased sound sensitivity	Scene analysis, localization, attention, melody contour, accents, environmental sound recognition, working memory	Auditory stream separation, sound localization/motion detection^i^, DLT^1^^,2,3,4^	Posterior cingulate, precuneus, lateral temporo-parietal cortex
PCA^d^	Visuo-perceptual/visuo-spatial, other parietal deficits	Similar or more severe than typical AD	More severe involvement of auditory scene /spatial processing	Auditory stream separation, sound localization/motion detection^i^^,j,2,5 j^	
LPA^d^	Anomia, phonological and verbal working memory deficits	Similar or more severe than typical AD	Phoneme perception, prosody perception, working memory	Phoneme discrimination^j^^,6^	
LBD^e^	Fluctuating alertness/attention/executive deficits, visuo-perceptual deficits, visual hallucinations, REM sleep behaviour disorder, parkinsonism	Auditory hallucinations	Pure tone detection, complex tone perception, auditory scene analysis, rhythm perception, speech loudness perception	Sinewave speech comprehension^k^^,7,8^	Cortico-subcortical circuits
**FTD**					
nfvPPA	Speech production deficits, agrammatism	Agnosia for environmental sounds/accents, word deafness^f^	Pure tone detection, perception of pitch interval/timbre/rhythm/prosody, accent comprehension	Temporal pattern discrimination^9^	Peri-Sylvian networks, prefrontal cortex
svPPA	Anomia and vocabulary loss, visual agnosias, behavioural changes similar to bvFTD	Musicophilia/sound aversion^g^, tinnitus, phonagnosia/nonverbal sound agnosia	Environmental sound/voice recognition, emotional recognition/reactivity, hedonic valuation, integration of semantic/affective information	Environmental sound recognition^10^	Auditory/multimodal association cortex in anterior temporal lobe, orbitofrontal cortex, insula
bvFTD	Socio-emotional, executive dysfunction with disinhibition, apathy, loss of empathy, obsessions and rituals, dietary and other behavioural abnormalities	Sound aversion/musicophilia^g^, phonagnosia^h^	Emotional recognition/reactivity, hedonic valuation, voice recognition^f^, integration of semantic/affective information	Vocal emotion recognition^11^	Auditory/multimodal association cortex in anterior temporal lobe, orbitofrontal cortex, insula, anterior cingulate, striatal circuits

**Figure 3:**
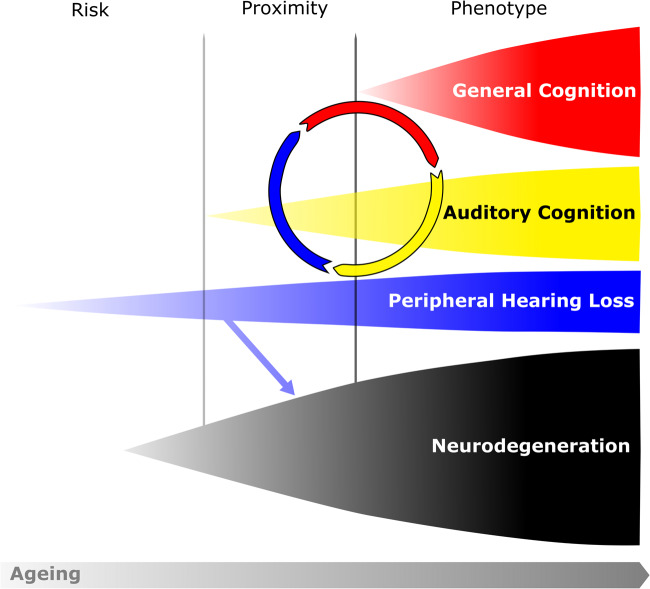
**A pathophysiological synthesis of hearing impairment and dementia.** This figure schematizes proposed relations between development of peripheral hearing loss (blue), changes in auditory cognition (gold) and general cognitive function (red) and underlying neurodegeneration (black), based on emerging epidemiological and pathophysiological evidence. Hearing loss can be considered a potential causal risk factor for cognitive decline (Risk), a proximity marker for incipient dementia (Proximity) or a feature of the established dementia syndrome (Phenotype), according to the time window in which it occurs; the mechanisms of these effects are distinct but likely to be interdependent. Alzheimer’s disease has been the major focus of epidemiological studies assessing the risk of developing dementia in association with hearing loss (Lin *et al.*, 2011; Taljaard *et al.*, 2016; Livingston *et al.*, 2017; Loughrey *et al.*, 2018), though the distinction from cerebrovascular and other pathologies is problematic; midlife hearing loss may account for ∼10% of all cases of dementia, and has been proposed to have a direct potentiating effect (arrow) on the evolution of neurodegeneration. Though the mechanism of this linkage is unclear, animal models suggest it could occur via cellular effects such as oxidative stress or altered gene expression (Frenzilli *et al.*, 2017; Park *et al.*, 2018), changes in neural circuit function (Oxtoby *et al.*, 2017; Bidelman *et al.*, 2019) or a complex interaction between aberrant circuit activity and protein spread (Griffiths *et al*., 2020). However, a direct causal effect has not been established: for example, peripheral hearing function was not associated with brain amyloid deposition (a relatively specific preclinical marker of Alzheimer’s disease) in a large cohort of cognitively healthy older people (Parker *et al.*, 2020) and such an effect would still not account for the majority of cases of dementia with hearing alterations. Here we suggest that alterations in ‘central’ hearing or auditory cognition may constitute an early warning signal of incipient dementia, due to the computational demands imposed by listening in challenging everyday acoustic environments. In support of this idea, predominantly central auditory deficits (involving, for example, dichotic listening) have been shown to predict CSF tau levels and regional atrophy profiles consistent with Alzheimer’s disease pathology in cross-sectional studies (Tuwaig *et al*., 2017) and longitudinal development of a clinical syndrome compatible with Alzheimer’s disease (Gates *et al.*, 2011), while large genetic and neuropathological surveys have suggested changes in hearing (in particular, speech-in-noise perception) may be a preclinical marker of neurodegeneration (Brenowitz *et al.*, 2020*a*, *b*). We emphasize that deficits of peripheral and central hearing and more general cognitive functions are likely to interact strongly, with ‘vicious cycling’.

The table summarizes major clinical features, and auditory cognitive deficits, candidate auditory cognitive tests for early diagnosis and neuroanatomical associations in canonical dementia syndromes for which adequate data are available (see also Fig. 3).

^a^ Auditory domains affected based on behavioural test performance;

^b^ Based currently on experimental studies (examples referenced below) with a view (particularly for Alzheimer’s disease) to potential scalability, e.g. online administration, but provisional and require further clinical validation;

^c^ Major distribution of pathological changes in brain networks relevant to auditory deficits, as assessed using voxel-based morphometry, functional neuroimaging (chiefly functional MRI) and/or post-mortem material;

^d^ Underpinned by Alzheimer pathology in majority of cases;

^e^ Includes dementia with Lewy bodies and Parkinson’s disease dementia;

^f^ Not usually severe;

^g^ Associated with altered autonomic responses to sound;

^h^ Particularly associated with right temporal lobe atrophy;

^i^ Can be delivered via headphones using virtual space stimuli;

^j^ Other auditory abnormalities analogous to typical Alzheimer’s disease;

^k^ Processing of degraded (e.g. sinewave-transformed) speech that is subject to perceptual learning and modulated by neurotransmitter function, by analogy with tests on degraded visual stimuli that show promise for diagnosis of LBD.

AD = Alzheimer’s disease; bvFTD = behavioural variant frontotemporal dementia; DLT = dichotic listening test; FTD = frontotemporal dementia; LBD = Lewy body disease; LPA = logopenic aphasia; nfvPPA = non-fluent agrammatic variant of progressive non-fluent aphasia; PCA = posterior cortical atrophy; svPPA = semantic variant of primary progressive aphasia.

Examples of experimental studies using proposed tests:

^1^ Goll *et al.*, 2012*a*;

^2^ Golden *et al.*, 2015*c*;

^3^ Tuwaig *et al.*, 2017;

^4^ Gates *et al.*, 2011;

^5^ Hardy *et al.*, 2020;

^6^ Johnson *et al.*, 2020;

^7^ Weil *et al*., 2017;

^8^ Hardy *et al.*, 2017*c*;

^9^ Grube *et al.*, 2016;

^10^ Golden *et al.*, 2015*b*;

^11^ Omar *et al.*, 2011.

### Alzheimer’s disease

Alzheimer’s disease produces a core impairment of auditory scene analysis, not attributable to more elementary deficits of sound perception or generic cognitive capacities (Idrizbegovic *et al.*, 2011). Auditory scene processing deficits may predate onset of more generalized cognitive decline in people at risk of developing Alzheimer’s disease (Golob *et al.*, 2009; Gates *et al.*, 2011) and in both the typical amnestic and posterior cortical (visuospatial) syndromic presentations of Alzheimer’s disease (Goll *et al.*, 2012*a*; Golden *et al.*, 2015*c*; Hardy *et al.*, 2020), suggesting that such deficits are a functional marker of Alzheimer’s disease pathology. This interpretation would corroborate neuroanatomical findings linking impaired auditory scene analysis to dysfunction and atrophy of the temporo-parietal ‘default mode’ network that is essential to Alzheimer’s disease pathogenesis (Warren *et al.*, 2012; Goll *et al.*, 2012*a*; Golden *et al.*, 2015*a*, *c*) (Fig. 2).

More generally, auditory phenotypic features of Alzheimer’s disease may signify a unifying deficit in encoding sound sources and patterns as distinct auditory objects (Griffiths and Warren, 2004; Goll *et al.*, 2010*b*, 2011; Hailstone *et al.*, 2012; Hardy *et al.*, 2017*b*). Such a deficit might ultimately underpin environmental sound agnosia in Alzheimer’s disease (Coebergh *et al.*, 2020) and impaired phonological processing (most saliently in the logopenic variant) (Johnson *et al.*, 2020), amplified by abnormalities of auditory working memory (Dhanjal *et al.*, 2013).

### Lewy body disease

Auditory dysfunction is prevalent in the Lewy body disease (LBD) spectrum (Parkinson’s disease and dementia with Lewy bodies) and may be a marker of disease onset, evolution and severity (Seifan *et al.*, 2019; Jafari *et al.*, 2020). Diverse auditory phenomena have been reported, ranging from auditory hallucinations to impairments of auditory scene analysis, tone and rhythm processing (Mollaei *et al.*, 2019; Cochen De Cock *et al.*, 2020; Jafari *et al.*, 2020). Electrophysiologically, there is evidence of impaired auditory startle, deviance detection, habituation and sensory filtering (Perriol, 2005; Jafari *et al.*, 2020) as well as olivocochlear efferent pathway dysfunction (De Keyser *et al.*, 2019). The unifying deficit may be dynamic disruption of synaptic transmission at multiple levels of the auditory hierarchy (Jafari *et al.*, 2020), due to abnormal top-down, neuromodulatory (principally dopaminergic) regulation.

### Frontotemporal dementias

Auditory perceptual dysfunction is emerging as a core feature of nfvPPA (Goll *et al.*, 2010*a*, 2011; Golden *et al.*, 2016; Grube *et al.*, 2016; Hardy *et al.*, 2019), including deficits of rhythm, pitch and timbre perception (Goll *et al.*, 2010*a*, 2011; Grube *et al.*, 2016) and sound detection (Hardy *et al.*, 2019). The key mechanism is likely to be impaired auditory pattern analysis in peri-Sylvian and connected prefrontal regions that govern expectations about incoming sensory traffic (Cope *et al.*, 2017; Hardy *et al.*, 2017*a*, *b*) (Fig. 2).

In contrast, semantic variant PPA typically spares elementary auditory pattern perception, leading instead to degraded semantic analysis of environmental sounds, voices and affective auditory signals (Bozeat *et al.*, 2000; Goll *et al.*, 2010*a*, *b*, 2012*b*; Hailstone *et al.*, 2011; Fletcher *et al.*, 2015; Golden *et al.*, 2015*b*; Muhammed *et al.*, 2018). This profile reflects selective degeneration and functional reorganization of antero-medial temporal lobe (Fig. 2) and its connections, including orbitofrontal cortices and auditory thalamus.

In the behavioural variant of frontotemporal dementia, inappropriate emotional reactions to voices, environmental sounds and music are often prominent (Omar *et al.*, 2011; Fletcher *et al.*, 2015): these are likely to be driven by impaired valuation and regularity decoding in complex auditory environments, linked to dysfunction of neural circuits mediating reward and rule processing (Clark *et al.*, 2017, 2018).

## Hearing impairment: cause, canary or corollary of dementia?

The complex pathophysiological relations between hearing impairment and dementia (schematized in Fig. 3) remain to be fully defined. Impoverished sensory fidelity due to peripheral hearing loss or disturbed subcortical auditory trafficking will potentially have effects both on auditory cognition and more general cognitive functions such as attention, executive processing and perceptual learning (Loughrey *et al.*, 2018; Fig. 1), leading to ‘vicious cycling’. Hearing loss might therefore produce both syndromic and generic cognitive signatures. The balance of these is likely to depend on stimulus and task demands as well as the particular neurodegenerative process. Emerging epidemiological evidence suggests that hearing impairment may potentiate neurodegeneration, perhaps via an interaction of aberrant auditory activity with culprit proteinopathies in vulnerable neural circuits (Griffiths *et al.*, 2020) (Fig. 3). Indeed, hearing impairment might constitute a facilitating cause of neurodegenerative disease evolution, an early warning ‘canary’ for impending cognitive disaster or an accompaniment of established dementia: these non-exclusive mechanisms would have mutually reinforcing consequences for auditory brain function.

## Conclusions: a synthesis and future view

The balance of neuroanatomical, physiological and clinical evidence suggests that the auditory brain plays a key role in the increasingly well documented association between dementia and hearing impairment. Degeneration of central auditory processing mechanisms (in particular, auditory cognitive dysfunction) will tend to amplify any degree of peripheral deafness and reduce compensatory capacity under natural (noisy) listening conditions. This reflects the extensive reciprocal interplay between afferent and efferent auditory processing pathways, exquisitely vulnerable to neurodegenerative proteinopathies. Moreover, neurodegenerative pathologies have distinct and relatively specific auditory cognitive phenotypes as well as generic effects on cognitive functions relevant to hearing, in line with the large-scale neural network signatures of these diseases. The synthesis we propose has neurobiological, diagnostic and management implications that should be tackled in future work.

Neurobiologically, central auditory dysfunction is likely to be a fundamental, early consequence of neurodegenerative dementias, due both to direct involvement of susceptible auditory processing networks by pathogenic protein spread and remote effects on highly interconnected structures. This requires substantiation using physiologically grounded neuroimaging techniques such as functional MRI and magnetoencephalography that may also help clarify the neural mechanisms of compensatory and therapeutic effects. Detailed, longitudinal disease phenotyping with biomarker and ultimately histopathological support (accounting for healthy auditory ageing and comorbid disease) will be required to elucidate the auditory pathophysiological signatures of particular proteinopathies, to assess the relative importance of hearing impairment in different diseases and to clarify the role of peripheral hearing deficits in potentiating the neurodegenerative process (Griffiths *et al*., 2020).

Diagnostically, hearing impairment might plausibly constitute a proximity marker for incipient cognitive decline and dementia, reflecting the heavy computational demands that auditory signal processing imposes on failing neural circuits. If substantiated in longitudinal studies of at-risk populations, this would raise the exciting prospect of novel auditory ‘cognitive stress tests’ for detecting the early stages of neurodegeneration and identifying dynamic, physiological biomarkers of disease evolution, residual plasticity and therapeutic response (Hardy *et al.*, 2018). Such markers could represent red flags for targeting population-based screening and recruitment into dementia prevention trials from primary care settings and could be developed into ‘digital biomarkers’ that are highly scalable. For example, headphone-based tests of spatial hearing, degraded speech perception and dichotic listening could be performed online (Gates *et al.*, 2011; Golden *et al.*, 2015*c*). In addition, developing a toolkit of novel tests to quantify the relative contributions of peripheral and central auditory deficits would allow accurate characterization of auditory phenotypes in individual patients and could facilitate diagnosis of particular neurodegenerative pathologies (Table 1). It will be crucial to capture the real-world impact of central hearing impairment, which is likely to be more profound than would be predicted by the degree of any peripheral hearing loss.

Management approaches that focus solely on peripheral sound amplification are likely to be of limited efficacy for improving hearing function in dementia. There is a clear practical and pathophysiological motivation to address any potentially reversible component of peripheral hearing loss and ensuring compliance with hearing aids (Proctor *et al.*, 2020). Ultimately, however, the goal of management should be to minimize hearing-related disability in the complex listening environments of daily life—to treat the patient, not the audiogram or the neuropsychological test score. Personalized interventions directed to central auditory mechanisms such as ‘smart’ hearing aids (Koohi *et al.*, 2017), hearing-based behavioural therapies and auditory cognitive rehabilitation (Russo *et al.*, 2005) should be combined with education and environmental modification supported by a detailed assessment of functional disability. Pharmacological modulation of cholinergic and dopaminergic function to harness auditory plasticity has shown early promise in Alzheimer’s disease and LBD (Dhanjal *et al.*, 2013; Hardy *et al.*, 2017*c*; Jafari *et al.*, 2020): such approaches could herald a new era of physiologically informed, integrated management focusing on retained capacity rather than deficits and embracing both central and peripheral auditory impairment in dementia.

## Funding

The Dementia Research Centre is supported by Alzheimer’s Research UK, Brain Research Trust, and The Wolfson Foundation. This work was supported by the Alzheimer’s Society, Alzheimer’s Research UK and the National Institute for Health Research University College London Hospitals Biomedical Research Centre. J.C.S.J. is supported by an Association of British Neurologists Clinical Research Training Fellowship, funded by Guarantors of Brain. C.R.M. is supported by a grant from Bart’s Charity. R.S.W. is supported by a Wellcome Clinical Research Career Development Fellowship (201567/Z/16/Z). D.E.B. is supported by a BRC Hearing and Deafness grant. C.J.D.H. is supported by an Action on Hearing Loss-Dunhill Medical Trust Pauline Ashley Fellowship. J.D.W. receives grant support from Action on Hearing Loss, Alzheimer’s Research UK, Alzheimer’s Society, Guarantors of Brain, Brain Research UK, MRC, Wellcome Trust, and the Wolfson Foundation.

## Competing interests

The authors report no competing interests.
